# Adaptive Optimization Method for Prediction and Compensation of Thin-Walled Parts Machining Deformation Based on On-Machine Measurement

**DOI:** 10.3390/s24020613

**Published:** 2024-01-18

**Authors:** Long Wu, Aimin Wang, Kang Wang, Wenhao Xing, Baode Xu, Jiayu Zhang, Yuan Yu

**Affiliations:** 1 Digital Manufacturing Institute, Beijing Institute of Technology, Beijing 100081, China; 2 School of Mechanical and Electrical Engineering, Shandong Jianzhu University, Jinan 250101, China; 3 Beijing Xinghang Mechanical-Electric Equipment Co., Ltd., Beijing 100074, China; baodexu1988@163.com (B.X.); zjy191007@163.com (J.Z.);

**Keywords:** thin-walled parts, deformation prediction, surrogate stiffness model, on-machine measurement, compensation

## Abstract

Thin-walled aluminum alloy parts are widely used in the aerospace field because of their favorable characteristics that cater to various applications. However, they are easily deformed during milling, leading to a low pass rate of workpieces. On the basis of on-machine measurement (OMM) and surrogate stiffness models (SSMs), we developed an iterative optimization compensation method in this study to overcome the machining deformation of thin-walled parts. In the error compensation process, the time-varying factors of workpiece stiffness and the impact of prediction model errors were considered. First, we performed machining deformation simulation and information extraction on the key nodes of the machined surface, and an SSM containing the stiffness information of discrete nodes of each cutting layer was established. Subsequently, the machining errors were monitored through intermittent OMM to suppress the adverse impact of prediction model errors. Further, an interlayer correction coefficient was introduced in the compensation process to iteratively correct the prediction model error of each node in the SSM along the depth direction, and a correction coefficient between parts was introduced to realize the iterative correction of the prediction model for the same node position between different parts. Finally, the feasibility of the proposed method was verified through multiple sets of actual machining experiments on thin-walled parts with added pads.

## 1. Introduction

Thin-walled aluminum alloy parts are widely used in the aerospace field because of their high specific strength, lightweight, and good machinability. However, the local stiffness in the thin-wall areas of the parts is relatively low, and thus, they are prone to severe machining deformation. This effect further affects the machining accuracy. These issues are primarily addressed using two approaches, namely, error suppression and error compensation [1]. Error suppression reduces the machining error by improving the machine tool accuracy, increasing auxiliary support, or reducing the cutting parameters. Although these error suppression methods are favorable, the machining cost is relatively high. Error compensation primarily compensates the deviations generated in the milling process in advance on the tool path according to the predicted machining deformation. The compensation method does not require machine tools or auxiliary support equipment and has strong versatility. However, compensation strategies have a significant impact on the performance of machining results. The primary objective of this study was to investigate the compensation method for the machining deformation of thin-walled parts.

Existing compensation strategies for machining deformation of thin-walled parts are primarily divided into two categories, namely, simulation-based compensation and on-machine measurement (OMM)-based compensation.

## 2. Literature Review

### 2.1. Simulation-Based Prediction and Compensation of Machining Deformation

Recent advances in finite element simulation technology have led to significant improvements in the accuracy of machining deformation prediction models established by industrial finite element modeling (FEM) software. Hence, simulation-based machining deformation prediction and compensation techniques have been widely used in recent years.

Machining Deformation Prediction Method: Xi et al. [2] established the stiffness matrix of an in-process workpiece (IPW) according to the super-element method. They considered the impact of material removal on the overall stiffness matrix and proposed a thin-walled part machining deformation prediction model based on the aforementioned method. On the basis of the reciprocity theorem, Tang et al. [3] established a theoretical deformation equation model of thin-walled plates under linear loads and predicted the machining deformation of parts. Agarwal et al. [4] presented a flatness error estimation model for end milling of thin-walled planar parts, which estimated the flatness-related parameters on the basis of a combination of a mechanical force model, workpiece deformation model based on finite element analysis (FEA), and an algorithm based on particle swarm optimization.

Compensation Based on FEM Prediction: Ma et al. [5] predicted the clamping deformation of thin-walled parts through FEM and established the mapping relationship of the tool contact points on the tool path before and after deformation. On the basis of this relationship, the tool path of the parts was modified to avoid machining errors caused by the clamping deformation and springback of the thin-walled parts. Li et al. [6] integrated a tool deformation calculation model and error-flexible iterative compensation to analyze the impact of tool deformation on the precision of blade finishing milling. They compensated the tool deformation errors on the tool path using mirror compensation. Li et al. [7] proposed a deformation prediction model and error compensation strategy for side milling of thin-walled parts. When calculating the force-induced deformations, the compensation value of the previous machining position was used as the initial iteration value to calculate the machining errors at the position, improving computation efficiency. Si et al. [8] proposed an iterative compensation strategy for side milling of thin-walled parts, wherein they calculated the deformations of the tool and the workpiece using the cantilever beam theory and FEM, respectively. Their approach helped in reducing surface errors caused by tool/workpiece deformation during milling by modifying tool tip position and tool axis orientation.

Deformation Prediction and Compensation Based on Surrogate Models: Although the accuracy of the prediction model has increased with improvements in FEM, the computational complexity is also increasing significantly with the improvement of accuracy, causing low computational efficiency. Sun et al. [9] proposed a method for quick estimation of machining deformation of thin-walled parts using a hybrid surrogate model. They combined the response surface regression and Gaussian regression models to establish a three-level gradient hybrid surrogate model and calibrated and quantified the uncertain factors of the model. Li et al. [10] designed a thin-wall micro-milling deformation compensation device, which, combined with a mathematical model based on the Rayleigh–Ritz theory to calculate thin-wall deformation, can compensate for micro-straight thin-wall machining errors. Yang et al. [11] proposed a flexible grinding control method for low-stiffness parts to achieve uniform thickness of the removed material. The model considers the case of dual flexibility of the workpiece and the tool and is capable of compensating both thin-walled workpiece deformation and tool deformation at nominal tool offsets.

For the aforementioned simulation-based compensation methods, it is necessary to reconduct machining deformation simulation analysis when changing the tool or machining parameters used in the machining process, which makes FEM time consuming and inflexible in application. Moreover, the simulation-based machining deformation compensation methods cannot make feedback adjustments based on the machining results. It is an open-loop system. As a result, optimizing the simulation prediction model of subsequent machining according to the known machining error data is difficult.

### 2.2. Measurement-Based Compensation Methods

Compared to the deformation prediction based on simulation, it is favorable to obtain the error information of the workpieces by OMM. With the emergence of measuring heads and software that can be directly applied to computer numerical control (CNC) machine tools, OMM-based compensation methods have been extensively studied in recent years and are now widely used, although measurement-based compensation methods require additional measuring equipment.

Compensation Regardless of Time-Varying Factors: Zhang et al. [12] proposed an OMM-based comprehensive error compensation method for the contour and thickness of curved surface parts. A comprehensive constraint that considers both contour and thickness was established using OMM, wherein a new target outer surface was constructed. The comprehensive method compensates for both shape and thickness errors, and it subsequently improves the overall pass rate of workpieces. Fan et al. [13] proposed a data-driven method for the separation and compensation of machining errors wherein the spatial statistical analysis method is integrated to categorize the machining errors into systematic and process errors. Their method compensates for the corresponding errors by integrating tool path adjustment and cutting parameter optimization. Xiong et al. [14] presented a closed-loop error compensation method for robot side milling on complex curved surfaces based on in situ measurement, wherein the spatial statistical analysis is carried out through Moran’s I to separate systematic and random errors and the processing path is compensated through mirror compensation. Ge et al. [15] proposed an OMM-based comprehensive error compensation method for thin-walled parts. First, the machining errors were reconstructed using OMM, and a compensation model was established using triangulation-based cubic interpolation and machine learning algorithms. Finally, real-time error compensation was realized by constructing an external zero-drift compensation system for the machine tool.

Compensation with Regard to Time-Varying Factors: For thin-walled parts, as the material is removed, the stiffness of the part gradually decreases. Simple mirror compensation based on the errors in the previous layer often cannot satisfy the accuracy requirements. Thus, Guiassa et al. [16] established a part compliance prediction model with intermittent OMM data, and they applied the model to compensate for the multi-pass milling of thin-walled parts. This method considers the impact of a corresponding reduction in the stiffness of the parts as the material is removed, thus yielding a better compensation effect than mirror compensation. Zhao and Zheng et al. [17] considered the time-varying characteristics of cutting conditions for thin-walled parts during the material removal process, and they established an online first-order machining error compensation model for thin-walled parts, which is corrected using the OMM results of the current machining layer for the compensation processing of the subsequent layer. Ge et al. [18] considered not only the influence of the material removal effect but also the coupling effect between compensation value and workpiece deformation and proposed a compensation algorithm without iterative processing.

The commonly used mirror compensation approach in existing measurement-based compensation methods does not consider the stiffness variation during material removal; this shortcoming may have an adverse impact on the compensation accuracy. However, measurement-based compensation according to stiffness change requires multi-layer machining (for at least three processing layers), which is low in machining efficiency, insufficient in historical data utilization, and low in the optimization efficiency for workpieces machined in batches.

**Figure 1:**
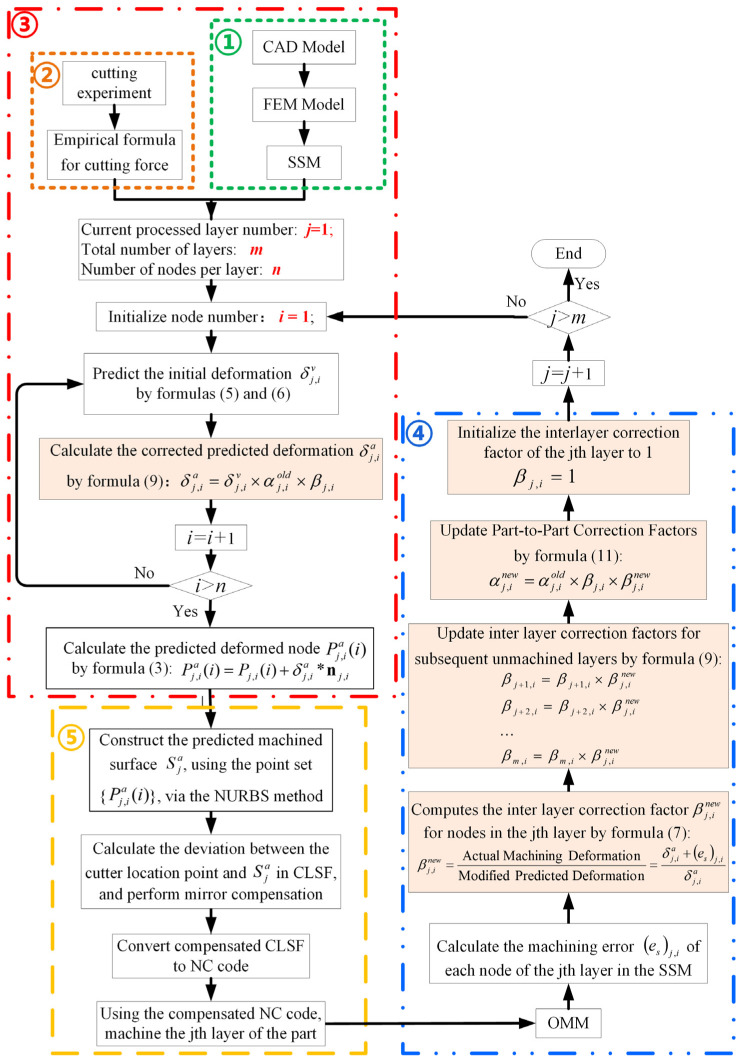
Iterative optimization compensation method for machining deformation incorporating OMM and SSM.

Considering the aforementioned problems, a fast prediction and iterative optimization compensation method of machining deformation that integrates OMM and surrogate stiffness models (SSMs) is proposed in this paper. The proposed method can combine the advantages of FEM and OMM methods and subsequently improve machining accuracy and efficiency. Figure 1 shows the compensation method, which includes five main modules: (1) SSM establishment (Section 3.1 and Section 3.2), (2) determination of the empirical formula of cutting force (Section 3.3), (3) prediction of machining deformation based on SSM (Section 3.4), (4) iterative correction of the prediction model with OMM and SSM (Section 4.1), and (5) compensation processing based on the revised prediction model (Section 4.2).

## 3. Efficient Machining Deformation Prediction Based on SSM

Many types of commercial finite element software can predict the machining deformation of parts before engineers carry out the machining of expensive large-scale thin-walled parts to avoid unnecessary machining deviations. These packages are, for example, Ansys, ABAQUS, Advantage, and SolidWorks [19]. But FEM model is generally inefficient in predicting the deformation of thin-walled parts, which is difficult to meet the demand for timely response and analysis in the machining process. In this chapter, we propose a fast machining deformation prediction method based on SSM, which can realize efficient machining deformation prediction of thin-walled parts.

### 3.1. Principle of SSM Establishment

**Figure 2:**
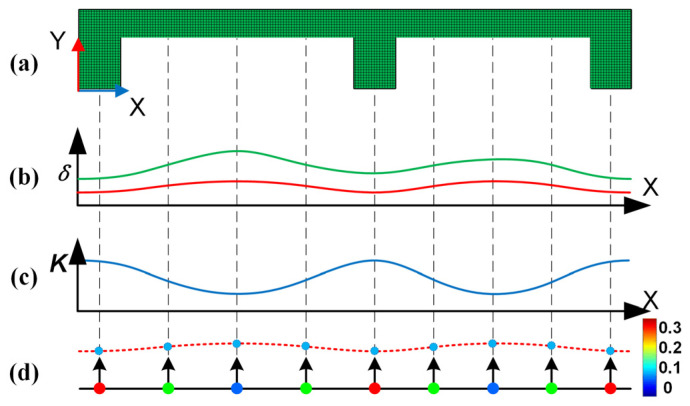
Process of machining deformation prediction based on discrete node information. (**a**) cross-sectional view of the workpiece, (**b**) the deformations under different cutting forces, (**c**) the local stiffness variation curve of the part, (**d**) the process of establishing stiffness model.

For the end milling of thin-walled parts, the finite element software ABAQUS was adopted to simulate the end milling process of the upper surface of the workpiece. Figure 2a shows a cross-sectional view of the workpiece. For the nodes on the upper surface, the same cutting force F, which is perpendicular to the surface, was applied from left to right in a specific sequence, and the deformation δ of each node caused by the force was recorded. The curve of workpiece deformation δ along the x direction was then established, as shown in Figure 2b, where the red and green curves represent the deformations under different cutting forces.

For the end milling of thin-walled parts, the finite element software ABAQUS 6.12-1 was adopted to simulate the end milling process of the upper surface of the workpiece. Figure 2a shows a cross-sectional view of the workpiece. For the nodes on the upper surface, the same cutting force *F*, which is perpendicular to the surface, was applied from left to right in a specific sequence, and the deformation *δ* of each node caused by the force was recorded. The curve of workpiece deformation *δ* along the x direction was then established, as shown in Figure 2b, where the red and green curves represent the deformations under different cutting forces.

Based on these simulation results, the local stiffness at each node position can be calculated using Hooke’s law, as follows:

*R_i_* = *F*/*δ_i_*, (1)

where *R_i_* and *δ_i_* are the local stiffness (N/mm) and deformation (mm), respectively, at the *i*-th node position.

The local stiffness of each position in the thin-walled part varies with the material and local structure of the workpiece in proximity. Within the elastic range of the workpiece, the local stiffness of a specific position on the part is relatively stable. For a smooth and continuous processing area, the local stiffness often varies continuously, wherein the local stiffness variation curve of the part can be established, as shown in Figure 2c. Therefore, the variation of machining errors caused by local stiffness changes is usually continuous. The surface after machining deformation can be represented by the smooth surface reconstructed by the predicted deformed nodes.

The specific establishment of the stiffness model is shown in Figure 2d. Here, the machining area was simplified into multiple spring components with different stiffness values. Among these, the local stiffness Ri at the node position is represented by the node colors, and the solid black line represents the target molding surface. The nodes $P_{0}(i)=\left(x_{0}\left(i\right),y_{0}\left(i\right),z_{0}\left(i\right)\right)$ that contain stiffness information are present on the target-forming surface. The black arrow represents the normal direction **n***_i_* at the node position. When the local stiffness *R_i_* and cutting force *F* of the node position are known, the machining deformation *δ_i_* of each node can be easily calculated using Equation (1).

In Figure 2d, the predicted node coordinates *P_d_*(*i*) after machining deformation are represented by light blue dots. It is obtained by moving the stiffness node along the normal direction for a deformation distance, and its calculation process is as follows:

(2) $$P_{d}(i)=P_{0}(i)+\delta_{i}\cdot n_{i}.$$

The smooth red dashed line reconstructed with *P_d_*(*i*) represents the predicted machined deformed surface. Thus, the local stiffness lattice carrying the local stiffness and position information of the parts can be used to represent the machining area and predict its deformation. In addition, it is important to note that node density affects the prediction accuracy of SSM, which we will discuss in the next section.

### 3.2. Structure and Composition of SSM

**Figure 3:**
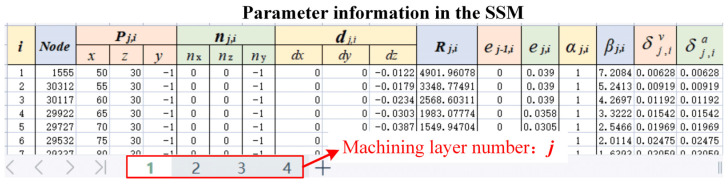
Structure and composition of SSM.

The stiffness information of the discrete nodes shown in Figure 2d can be fully expressed in a table. An SSM was built according to the format shown in Figure 3. The second column represents the node number that is used to store the node number information in the FEM software; columns 3–5 store the node coordinate values (*x*, *y*, *z*); columns 6–8 store the node surface vector (*nx*, *ny*, *nz*); columns 9–11 store the node deformation (*dx*, *dy*, *dz*) under the normal force calculated by the simulation software; and column 12 represents the local stiffness of the node positions along the normal direction that is calculated by the deformation and the normal force in simulation.

**Figure 4:**
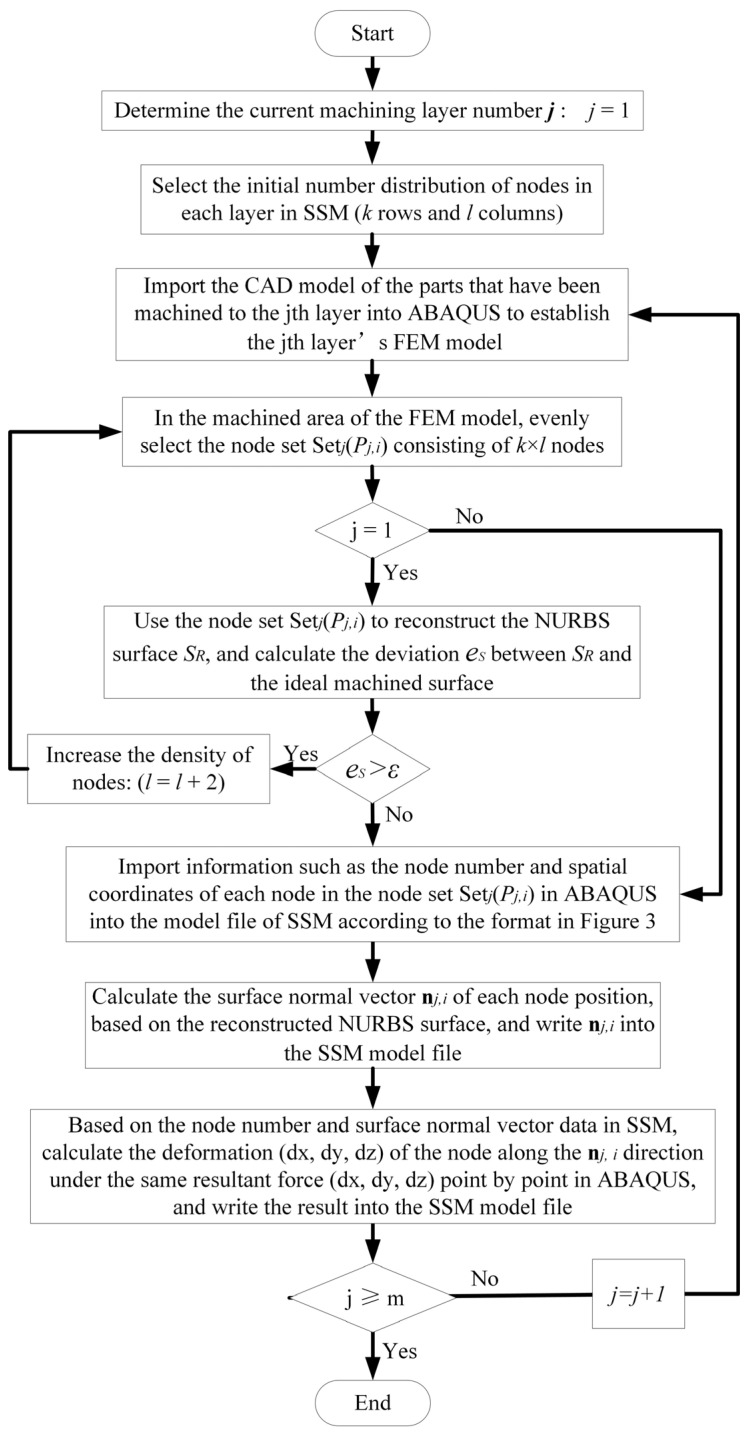
Process diagram for establishing the parametric stiffness model.

**Figure 5:**
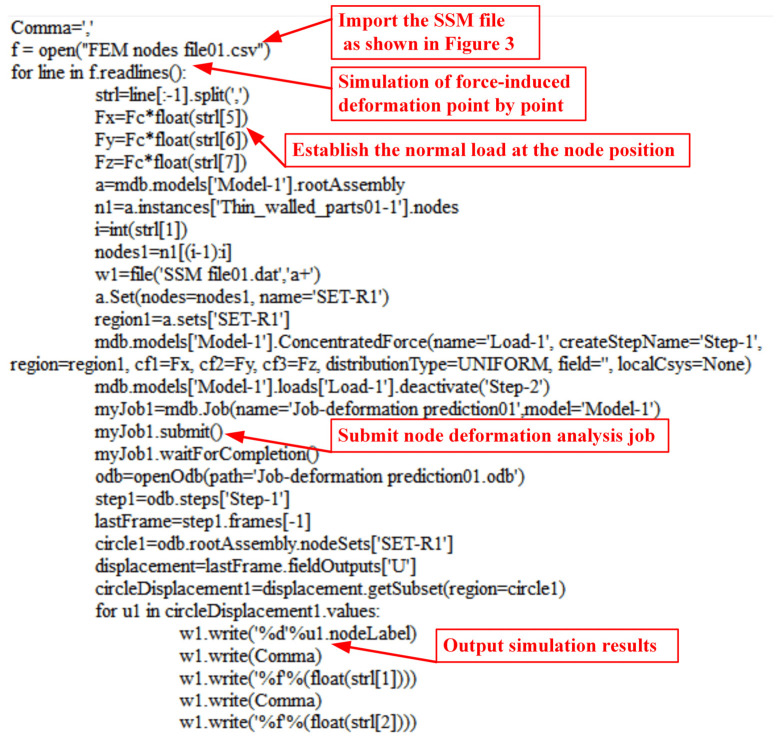
Automating simulation analysis and building the SSM in ABAQUS using scripts.

The content of columns 1–12 of the aforementioned SSM model can be automatically calculated and produced through further development of ABAQUS 6.12-1 software in Python. The specific process is shown in Figure 4. First, the machining layers should be determined. For workpieces that require multi-layer machining, an FEM model with multiple machining layers should be established, because it is more convenient to establish the process model of removing material layer by layer in a computer-aided design (CAD) model. Therefore, a FEM model representing the machining process of parts with different residual machining thicknesses was established using several CAD models. Notably, to ensure good SSM accuracy when reconstructing the machined surface, it is necessary to determine whether the deviation *e_s_* between the surface reconstructed by the node set *set*(***P_i_***) and the CAD model surface exceeds the maximum deviation limit *ɛ* when determining the density of the discrete nodes in SSM. If *e_s_* > *ɛ*, the node density in SSM should be increased until *e_s_* ≦ *ɛ*. After the node set of the machining surface *set*(***P_i_***) is determined, the node information is sequentially written into the first 5 columns of the SSM file in the format shown in Figure 3. The non-uniform rational B-spline (NURBS) surface was reconstructed using node sets *set*(***P_i_***), and the surface normal vector ni of each node was calculated from the reconstructed NURBS surfaces and written into columns 6–8 of the SSM. Using the script shown in Figure 5, the same force *F_c_* is applied one by one to the SSM nodes along the normal direction on the part model in ABAQUS. Then, the simulation analysis job is submitted in a cycle, and the force-induced deformation values of each node are extracted and recorded in columns 9–11 of the SSM. Finally, the local stiffness of the node is calculated using Equation (1) and written into column 12 of the SSM.

After establishing the SSM shown in Figure 3, the error calculation and compensation process can be applied independently from the finite element software. The SSM can continue to function even when machining parameters or cutting tools are changed, and only the empirical formula of the cutting force should be adjusted, which makes SSM more computationally efficient and flexible compared to conventional FEM. The data after column 11 in Figure 3 are used for SSM model correction based on OMM results, which is introduced in Section 3.

### 3.3. Cutting Force Estimation

An accurate estimation of the value of cutting force according to machining conditions is a prerequisite for predicting and compensating machining deformation for thin-walled parts. Significant research has been carried out on the prediction of cutting force in recent years. Chief among the methods are the mechanical method and the empirical method. The empirical method [20] considers that cutting force is related to cutting parameters and regards milling force as an exponential function of cutting parameters. The mechanical method [21] considers that cutting force is not only related to the cutting parameters but also to the tool geometry and other factors and regards cutting force as a function of chip area. In addition, in the actual machining of thin-walled parts, the dynamic characteristics of the tool and the workpiece affect the prediction and measurement of the milling force. Kiran et al. [22] proposed a modeling and accurate measurement method for cutting forces in flexible workpiece milling.

The mechanical force model is complicated, and the process of re-establishing the cutting force model after replacing the cutting tool places higher requirements on the theory and technology of the operator, which is not conducive to its application in industrial environments. The empirical approach is more direct and convenient. Therefore, the empirical method was used in this study to estimate the cutting force. According to the method mentioned in the literature [20], the empirical formula of cutting force is established as

(3) $$F=N\cdot C_{F}\cdot k_{F}\cdot a_{p}^{a}\cdot a_{e}^{b}\cdot f_{i}^{c}\cdot \Omega^{d},$$

where *a_p_*, *a_e_*, *f*, and *Ω* are cutting depth, cutting width, feed per tooth, and rotational speed, respectively; *N* is the number of tool teeth; *C_F_* is the cutting force coefficient; and *a*, *b*, *c*, and *d* are the exponential coefficients of the machining parameters, which can be calculated during cutting tests.

Notably, to ensure the accuracy of the empirical formula of the cutting force during machining, the center of the tool axis must be perpendicular to the workpiece surface when customizing the surface machining scheme.

### 3.4. Machining Deformation Prediction Considering Coupling between Force and Deformation

Once the empirical formula is established in the form of Equation (4) through cutting experiments, a predicted cutting force value can be obtained on the basis of a given set of machining parameters. However, the workpiece may be elastically deformed by the cutting force during machining, thereby reducing the actual cutting depth in end milling. This phenomenon, in turn, affects the cutting force. Therefore, force and deformation are in a coupling relationship [23]. In addition, the residual workpiece material (*e_s_*)*_j−_*_1,*i*_ of the previous step also affects the actual cutting force and depth.

**Figure 6:**
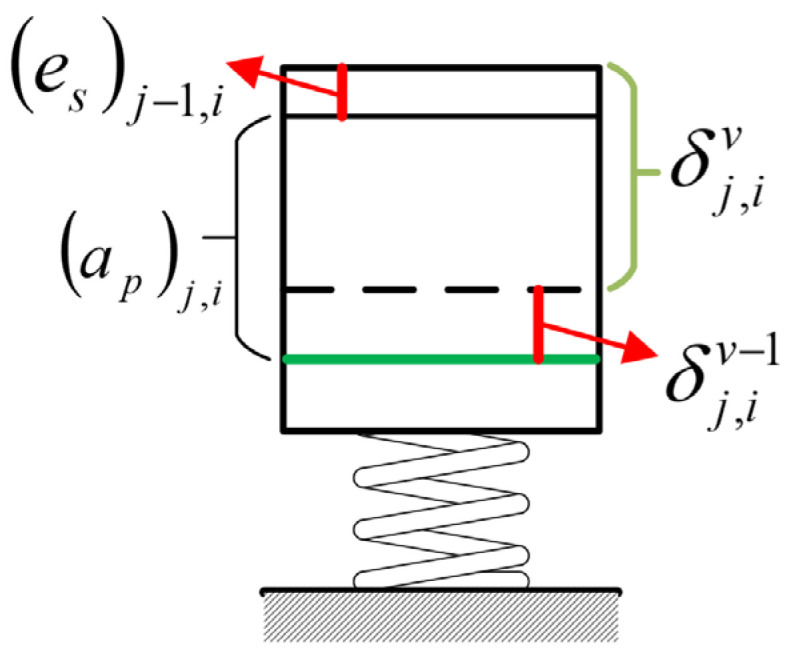
Coupling calculation of cutting force and deformation at stiffness node position.

Figure 6 shows a schematic of the actual cutting depth under the coupled iteration. In the figure, (*a_p_*)_*j*,*i*_ is the target cutting depth of the *i*-th stiffness node position in the *j*-th layer, with the subscript *j* in the parameter indicating the machining layer and *i* representing the node number; (*e_s_*)*_j−_*_1,*i*_, which can be calculated from OMM results, is the actual machining error of the *i*-th node position in the (*j* − 1)-th layer, and it also represents the residual thickness of the previous layer at the *i*-th node of the *j*-th layer. Further, $\delta_{j,i}^{v}$ is the deformation caused by the cutting force at the *i*-th node in the *j*-th layer, which is calculated in the *v*-th iteration. Figure 6 shows that the actual cutting depth under the influence of machining residual thickness (*e_s_*)*_j−_*_1,*i*_ and machining deformation can be expressed as $[{\left(a_{p}\right)}_{j,i}+{\left(e_{s}\right)}_{j-1,i}-\delta_{j,i}^{v-1}].$

The relationship between cutting force and workpiece deformation can be expressed in the subsequent iterative calculation formula:

(4) $$\delta_{j,i}^{v}=\frac{F\left[{\left(a_{p}\right)}_{j,i}+{\left(e_{s}\right)}_{j-1,i}-\delta_{j,i}^{v-1}\right]}{R_{j,i}},$$

where $F\left[{\left(a_{p}\right)}_{j,i}+{\left(e_{s}\right)}_{j-1,i}-\delta_{j,i}^{v-1}\right]$ is the cutting force that changes with cutting depth. Because the cutting parameters are known, cutting depth is the only variable that should be considered, and the cutting force can be regarded as a function of cutting depth. With $\delta_{j,i}^{0}=0$ as the initial value, the final stable cutting force and predicted deformation can be determined by the iterative calculations presented in Equation (4). The iteration termination condition can be expressed as

(5) $$\frac{\delta_{j,i}^{v}-\delta_{j,i}^{v-1}}{\delta_{j,i}^{v-1}}\leq \varepsilon ,$$

where *ɛ* is a minimal value that can be determined according to the actual situation.

Figure 3 shows that the coordinates of each node position in SSM, the surface normal vector, and the local stiffness value along the normal direction are known, and the empirical formula for cutting forces is also obtained. Further, the (*e_s_*)*_j−_*_1,*i*_ value of each node position can also be easily calculated from the OMM results. The predicted value of machining deformation of each node can be obtained using Equations (4) and (5) on the basis of the SSM calculation.

It should be noted that the initial predicted deformation $\delta_{j,i}^{v}$ is calculated from the SSM stiffness information, and this parameter should be corrected further to acquire the actual compensation $\delta_{j,i}^{a}$. The specific machining deformation prediction process is shown in box 3 in Figure 1. The model correction process is discussed in Section 3.1.

## 4. Iterative Optimization Compensation Method Based on OMM and SSM

In view of the problem that the existing compensation methods based on prediction models cannot be adjusted according to the measured results, in this paper, we propose a compensation method that combines OMM and SSM. The established prediction model can be iteratively optimized according to the measured results.

### 4.1. SSM Iterative Optimization Based on OMM

An SSM that is capable of flexible and efficient machining deformation prediction was established as described in the previous section. However, a machining deformation prediction and compensation system that depends solely on SSM is still an open-loop system, and it cannot optimize the subsequent compensation process based on the error information generated during machining. To realize closed-loop control of the deformation compensation system and subsequently improve the precision of end milling of thin-walled parts, this paper proposes an iterative optimization compensation method by incorporating OMM and SSM.

It should be noted that improper OMM schemes can introduce significant errors, especially in the arrangement of sampling points, because the accuracy of the substitute geometric model reconstructed using the measurement results is positively related to the final compensation accuracy. Therefore, it is necessary to optimize the sampling scheme and evaluate its reconstruction accuracy before conducting compensation machining based on OMM [24]. Sampling optimization methods have already been discussed in our prior works [24]; therefore, this article does not discuss them in depth. This paper focuses on the compensation method of machining deformation.

According to the definition of detection technology in the machining process in the literature [25], the detection method used in this study belongs to the category of contact OMM after processing. The iterative optimization compensation machining combined with OMM and SSM is an intermittent on-machine compensation method based on the measured results after processing.

**Figure 7:**
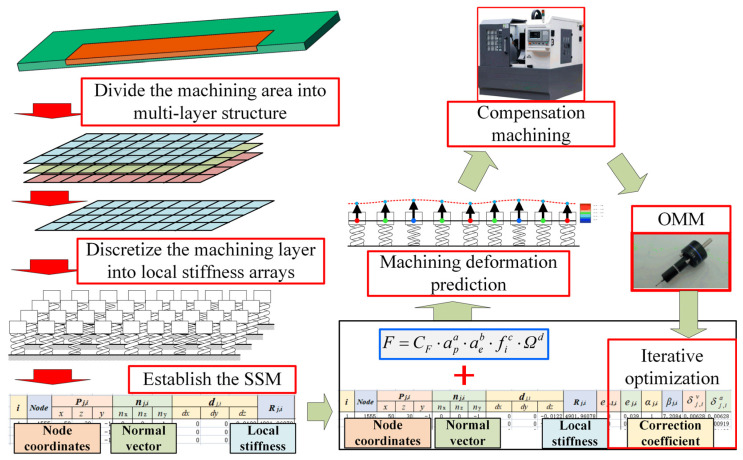
Iterative optimization compensation method combining SSM and OMM.

The process of establishing SSM is to simplify the FEM model into a certain number of particle spring models including the normal information of the node surface and the local stiffness information. As shown in Figure 7, after the SSM is established, it contains the stiffness information of each node on the machined surface. It can predict and compensate for the machining deformation directly without FEM software. This approach can improve the efficiency of error analysis and reduce downtime during workpiece machining. In addition, combined with OMM technology, the prediction deviation data of discrete nodes of SSM can be obtained intermittently. From these data, the local stiffness model of each node of SSM can be modified specifically to improve the accuracy of subsequent deformation prediction.

**Figure 8:**
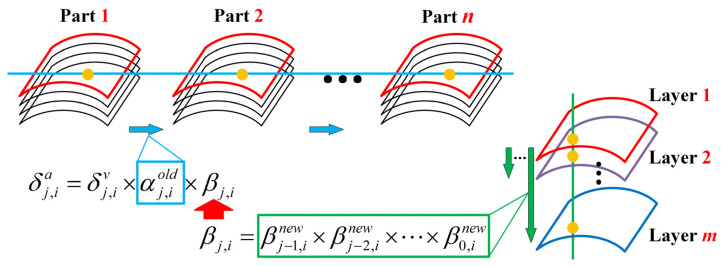
Iterative update process of *α*_*j*,*i*_ and *β*_*j*,*i*_ based on OMM.

As described in Section 2.1, the machining area is discretized into a set of mutually independent node arrays, wherein each node is considered a spring component, and their local stiffness, i.e., the elastic force of the spring, is fixed. Therefore, as shown in Figure 8, for the same type of workpieces, from part 1 to part *n*, the model error at the same node position in the SSM of the workpiece is approximately the same. Therefore, the prediction model error of the *i*-th node position in the *j*-th layer of the previous part in the SSM can be used to calculate the inter-part correction coefficient *α*_*j*,*i*_ at the node in the SSM to correct the prediction model for the same nodes in the SSM of the current part.

For the same workpiece, the thickness of the removed material is relatively small compared to that of the remaining part because of the layer-by-layer machining. Therefore, we can consider that the local stiffness values of nodes at the same position on different cutting layers change continuously along the depth direction of the machined surface of the part. This phenomenon can be expressed as follows: from layer-1 to layer-*m*, as the material is removed layer by layer, the local stiffness value of the *i*-th node position in the SSM gradually decreases. Further, the prediction model error of the *i*-th node in the previous layer ((*j* − 1)-th layer) of the SSM can be used to calculate the interlayer correction coefficient *β_j_*_−1,*i*_, which is used to correct the prediction model of the *i*-th node of the current layer (*j*-th layer). Differences in machining deformation tendency may exist between parts of the same batch because of the influence of local structural differences and local material distribution uniformity. The interlayer correction coefficient *β_j_*_−1,*i*_ introduced in this study can promptly correct the differences between parts.

To implement the aforementioned iterative optimization process in SSM, six columns of parameters were added in the SSM. As shown in Figure 3, the added parameters were (*e_s_*)_*j*−1,*i*_, (*e_s_*)_*j*,*i*_, $\alpha_{j,i}$, $\beta_{j,i}$, $\delta_{j,i}^{v}$, and $\delta_{j,i}^{a}$.

To conveniently show the change of parameters in the update process, the correction coefficient of the previous part before iteration correction is represented by $\alpha_{j,i}^{old}$ and $\beta_{j,i}^{old}$ with superscript; here, $\alpha_{j,i}^{new}$ and $\beta_{j,i}^{new}$ denote the correction coefficients that are updated according to the OMM results during the current part machining process. The specific iterative optimization process is shown in box 4 in Figure 1. Notably, all $\beta_{j,i}^{old}=1$ initial values in SSM are 1. The calculation method of each layer’s $\beta_{j,i}^{new}$ value is shown in Equation (6). It must be calculated by the machining error of the *i*-th node of the current layer measured by OMM after the compensation machining of the current layer (the *j*-th layer) is completed.

Each layer should be calculated from the machining error of the *i*-th node in the current layer measured by the OMM after the compensation of the current layer (*j*-th layer).

(6) $$\beta_{j,i}^{new}=\frac{\mathrm{Actual}\;\mathrm{Machining}\;\mathrm{Deformation}}{\mathrm{Modified}\;\mathrm{Predicted}\;\mathrm{Deformation}}=\frac{\delta_{j,i}^{a}+{\left(e_{s}\right)}_{j,i}}{\delta_{j,i}^{a}},$$

where ${\left(e_{s}\right)}_{j,i}$ is the machining error measured by OMM after the compensation machining of the current layer (layer *j*) is completed. $\delta_{j,i}^{a}$ is the actual compensation in the process that, as shown in box 3 in Figure 1, was obtained from the correction of the initial machining deformation amount $\delta_{j,i}^{v}$ predicted in SSM. This value was calculated before the compensation machining, and its correction process can be expressed as

(7) $$\delta_{j,i}^{a}=\delta_{j,i}^{v}\times \alpha_{j,i}^{old}\times \beta_{j,i},$$

where $\alpha_{j,i}^{old}$ is the inter-part correction coefficient of the previous part at the current node position (the *i*-th node in the *j*-th layer), and $\beta_{j,i}$ is the interlayer correction coefficient that is updated in a layer-by-layer manner with the deepening of the cutting layer. The updating process of $\beta_{j,i}$ in SSM is shown in box 4 in Figure 1. After the calculation of $\beta_{j,i}$ at the current node is completed, the interlayer correction coefficient of the node at the same position in the subsequent unprocessed layer in SSM is also updated. The calculation process is shown in Equation (8), where *m* is the total number of machining layers.

(8) $$\begin{array}{l} \beta_{j+1,i}=\beta_{j+1,i}\times \beta_{j,i}^{new}\\ \beta_{j+2,i}=\beta_{j+2,i}\times \beta_{j,i}^{new}\\ \ldots \\ \beta_{m,i}=\beta_{m,i}\times \beta_{j,i}^{new} \end{array},$$

From the first to the *j*-th layer, the interlayer correction coefficient $\beta_{j,i}$ can be expressed as the product of the interlayer correction coefficient of the processed cutting layers after the iterative update. For consistency in the structure of the formula, it is assumed that $\beta_{0,i}^{new}$ is present, and $\beta_{0,i}^{new}=1$. Hence, $\beta_{1,i}=\beta_{0,i}^{new}=1$ and $\beta_{j,i}$ can be expressed as

(9) $$\beta_{j,i}=\beta_{j,i}^{old}\times \beta_{j-1,i}^{new}\times \beta_{j-2,i}^{new}\times \cdots \times \beta_{0,i}^{new}.$$

As shown in Equation (7), $\alpha_{j,i}^{old}\times \beta_{j,i}$ was applied to correct the current node prediction model, where $\beta_{j,i}^{new}$ is the interlayer correction coefficient of the current layer calculated from the compensated machining error ${\left(e_{s}\right)}_{j,i}$. Therefore, we can use $\beta_{j,i}^{new}$ to re-correct the correction factor $\alpha_{j,i}^{old}\times \beta_{j,i}$ in the compensation process and assign the values to $\alpha_{j,i}$ for the prediction model correction at the same position of the next part. This process can be expressed as

(10) $$\alpha_{j,i}^{new}=\alpha_{j,i}^{old}\times \beta_{j,i}\times \beta_{j,i}^{new}.$$

After $\alpha_{j,i}$ is updated, $\beta_{j,i}$ at the current node is reinitialized to 1 for updating the next part. On the basis of the aforementioned iterative update method of the correction coefficient, we can conveniently and efficiently complete the iterative correction of the prediction model from the OMM results.

### 4.2. Machining Deformation Compensation Based on the Optimized Stiffness Model

**Figure 9:**
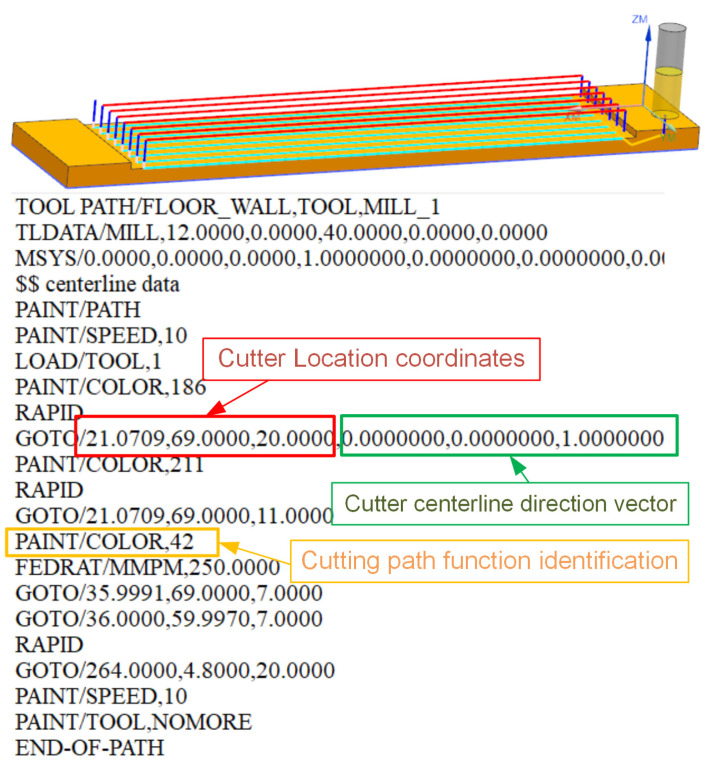
Tool path and CLSF file parameter identification.

In the previous section, we explained the OMM-based iterative correction method for SSM. In this section, we introduce the method for compensation machining according to the corrected deformation prediction results. In this study, the compensation of machining deformation was achieved through the identification and modification of the cutter location source file (CLSF). As shown in Figure 9, the CLSF contains information such as cutter location coordinates, tool centerline direction, and tool path function parameters. During end milling, the tool centerline is always perpendicular to the part surface. Therefore, according to the principle of mirror compensation, for the deformation compensation of the end milling process, we need only translate the cutter location coordinates along the direction of the tool centerline by a distance that is equal to the predicted deformation.

However, in the specific error compensation process, the following two points merit special attention: (1) if there is a long straight-line segment trajectory on the tool path, cutter locations should be added according to their corresponding distance; and (2) when calculating cutter location error, error calculation should be performed on the basis of the reconstructed model if the cutter locations do not overlap the measurement points or stiffness nodes.

#### 4.2.1. Machining of Long Straight Segment Trajectories

**Figure 10:**
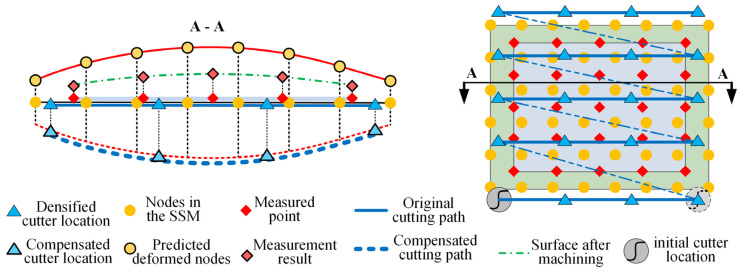
Error calculation and compensation process of stiffness model.

For the longer straight segment trajectories on the tool path, generally, only the start and end points of the straight segment trajectories are marked in the CLSF. However, for thin-walled parts, the machining deformation on the straight trajectory may not be uniform, and it may vary significantly according to local stiffness on the trajectory. As shown in the cross-section curve in Figure 10, the predicted machining deformation cross-section line was a bow curve, although the ideal tool path is a straight line. With regard to the deformation shown in Figure 10, if compensation is performed according to the original machining scheme, and error calculation and compensation are realized only on both ends of the straight line segment, the requirements of machining accuracy would not be satisfied.

To address the aforementioned issue that the long straight line segment trajectory cannot be compensated, the distance between the cutter locations is restricted in the CLSF file and the maximum distance between the tool points is 3 mm. Before the compensation of the tool path, the CLSF file was preprocessed, the cutter locations on the tool path were identified, and the distance between the cutter locations was evaluated. For straight-line segments that are larger than the set spacing requirements, cutter locations were added evenly until the requirements were met.

#### 4.2.2. Error Calculation and Compensation Process of the Stiffness Model

For the iterative correction process of the prediction model, the machining error ${\left(e_{s}\right)}_{j,i}$ of each node position in the SSM must be calculated. For the compensation process of the tool path, an accurate calculation of the predicted machining deformation error *e_t_* of each cutter location is necessary. However, as shown in the vertical view of the machining area on the right in Figure 10, the cutter locations, stiffness nodes, and measurement points did not coincide. Therefore, these two kinds of errors cannot be obtained directly through measurement. While calculating the errors, the nodes in each layer of the SSM and the measurement points were reconstructed into parametric surfaces, respectively, and the deviations at the corresponding positions were calculated accordingly. For the reconstruction of parametric surfaces, we resort to the NURBS method.

A NURBS surface of degree *k × l* can be expressed in the form of the following rational fraction [26]:

(11) $$S\left(u,v\right)=\frac{\sum_{i=0}^{m}\sum_{j=0}^{n}\omega_{ij}d_{ij}N_{i,k}\left(u\right),N_{j,l}\left(v\right)}{\sum_{i=0}^{m}\sum_{j=0}^{n}\omega_{ij}N_{i,k}\left(u\right),N_{j,l}\left(v\right)},\;0\leq u,\;v\leq 1,$$

where ***d****_ij_* is the control vertex, which is distributed in a rectangular array; *m* and *n* are the numbers of control points in the *u* and *v* directions, respectively; and *ω_ij_* is the weight factor corresponding to ***d****_ij_*, N_*i*,*k*_(*u*) is a basis function of degree *k* in the *u* direction, and N_*j*,*l*_(*v*) is a basis function of degree *l* in the *v* direction. The basis functions of these two directions were calculated from the sum of node vectors $U=[u_{0},u_{1},\cdots ,u_{m+k+1}]$ and $V=[v_{0},v_{1},\cdots ,v_{n+l+1}]$ in the *u* and the *v* directions, respectively, according to the de Boer recursive formula.

(*e_s_*)*_j_*_,*i*_ is the machining error of the position of the *i*-th node in the *j*-th layer in the SSM. It is the distance from the node along the surface normal direction to the intersection point of the NURBS surface reconstructed from the measured points. Here, the node coordinates and the surface normal vector of the node position were known from SSM. Similarly, *e_t_* is the distance from the cutter location along the vector direction of the tool centerline to the intersection point of the reconstructed predicted deformation surface, when calculating the predicted deformation of the tool position point *e_t_*, and the tool position coordinates and tool centerline vector were known through CLSF. The calculation process of these two errors is the same and both are aimed at evaluating the distance from the starting point of the ray to the intersection point of the ray and the parametrical surface according to the known starting point and direction of the ray. The numerical method mentioned in our previous research [22] was applied to perform the calculation process. This process is explained as follows. The ray was spatially transformed together with the NURBS surface so that the ray coincides with the Z-axis. At this time, the projection of the intersection point on the XOY plane was zero. The projection plane XOY can be divided into four parts using the bisection method along the parameters *u* and *v* directions. Then, according to the zero point on the projection plane XOY, the parameter interval [(*u_a_*, *u_b_*), (*v_a_*, *v_b_*)] where the intersection point is located can be quickly found. Repeating this dichotomy narrows the parameter interval where the intersection point lies. The intersection coordinates could be represented as (*u_a_*, *u_b_*) when the parameter-l interval is sufficiently small. Finally, the desired intersection coordinates and corresponding distance could be obtained by inputting the corresponding parameter values (*u_a_*, *u_b_*) into the NURBS curve equation before the space transformation.

As shown in Figure 10, because of the restriction associated with the size of the probe, the measurement points could not cover all the machining areas, contrary to the nodes in SSM. When calculating the prediction deviation of the nodes in SSM, some nodes in the boundary area would exceed the range of the measurement area; in this case, we found the nearest point to the SSM node on the four outer curves of the surface that is reconstructed from the measurement points. We used the machining error of this point as the prediction error of the SSM node. A similar situation occurred in the calculation of the tool position point error where some tool position points were not within the machining surface. However, because it overlapped with the coverage area of the tool machining, we ignored the tool position points that were not present in the machining area, without performing compensation.

## 5. Case Study

This section describes the experiment we conducted to verify the effectiveness of the proposed iterative optimization compensation method combining OMM and SSM. We considered thin aluminum alloy plates as an example for the verification test of end milling.

### 5.1. Experimental and Simulation Environment

**Figure 11:**
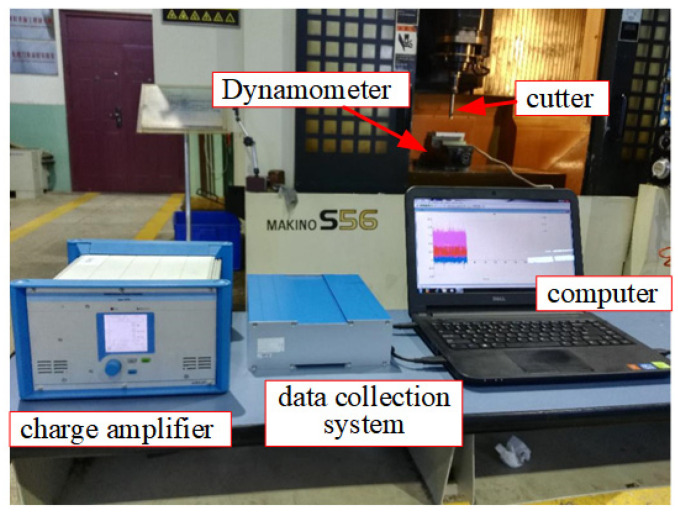
Machining environment and cutting force test.

The equipment adopted for the experimental process is shown in Figure 11. The Makino S56 vertical machining center was used for the machining in the experiment. The machining center was also equipped with an MP600 touch probe manufactured by Renishaw for on-machine inspection after machining. The cutting force measuring equipment was a Kistler 9272. The cutting force tests and the subsequent parts machining experiments were carried out on the same machine tool. The machining tool was a φ12 carbide end mill with four teeth.

According to the empirical method for cutting force estimation mentioned in the literature [20], the cutting force orthogonal tests were carried out according to different cutting parameters, and the parameters to be determined in the cutting force empirical formula (Equation (3)) were determined accordingly. In end milling, the deformation of thin-walled parts is mainly caused by the normal force *F_z_*. Therefore, only normal force was used in the deformation prediction analysis. The forces in the other direction are ignored. Equation (12) shows the empirical formula of normal force whose undetermined parameters were determined:

(12) $$F_{z}=4283.24a_{p}^{0.4213}a_{e}^{0.3355}n^{-0.6048}f^{0.3905}.$$

In the subsequent experiments related to compensation machining, the machining parameters were *a_p_* = 1 mm, *a_e_* = 5 mm, *n* = 3000 r/min, and *f* = 0.2 mm (feed per tooth).

**Figure 12:**
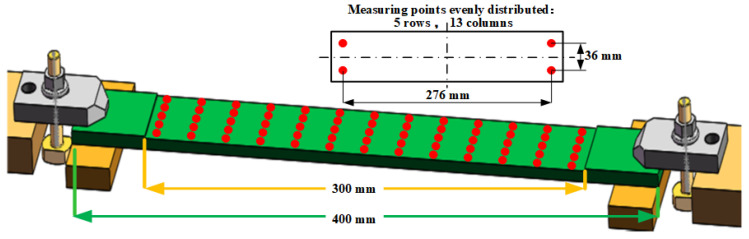
Test part structure and distribution of measurement points.

The thin plate part used in our experiments is shown in Figure 12. The dimension of the thin plate part was 400 × 40 × 6 mm, and it was clamped on both ends. The middle suspended area of size 300 × 40 mm was the machining area. The depth of each machining was 1 mm, and it comprised three layers. After the machining of every layer, the touch probe mounted on the machine tool conducted OMM. The sampling points, a uniformly distributed point array of 13 columns and 5 rows, are indicated by the red dots in the figure. The material used for the parts was AL6061; the material parameters are shown in Table 1.

**Figure 13:**
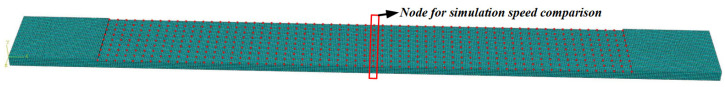
Selected nodes for establishing SSM in ABAQUS, and some nodes for computational efficiency comparison tests.

The SSM for the machining area of thin plate parts was established in the method introduced in Section 2.2. Figure 13 shows the distribution of the nodes selected in the finite element software ABAQUS 6.12-1 to construct the SSM. Because it was a plate part, a uniformly distributed 9 × 61 node array was chosen as the node set of one layer in SSM.

After determining the cutting force empirical formula of the part and establishing the SSM, the machining deformation in each machining layer of the part could be quickly predicted.

### 5.2. Comparison of Calculation Effects between SSM and Conventional FEM

This section provides a detailed comparison between the effects of the proposed SSM-based machining deformation prediction method and those of the traditional FEM. As shown in Figure 13, a group of nodes at the center of the thin plate was selected as the object to compare the computational efficiency and accuracy of the two methods.

The comparison was conducted on the same computer (CPU: Intel i5-1035G1; RAM: 16 GB). The finite element software adopted in the traditional FEM-based deformation prediction process was ABAQUS2016, which was further modified with Python to realize the automatic iterative calculation of the coupling between force and deformation. The proposed SSM-based method used a program that was independently developed with Python. Both methods consider the impact of the coupling between force and deformation, and the iteration termination condition in both cases was *ɛ* ≤ 0.01 mm.

**Figure 14:**
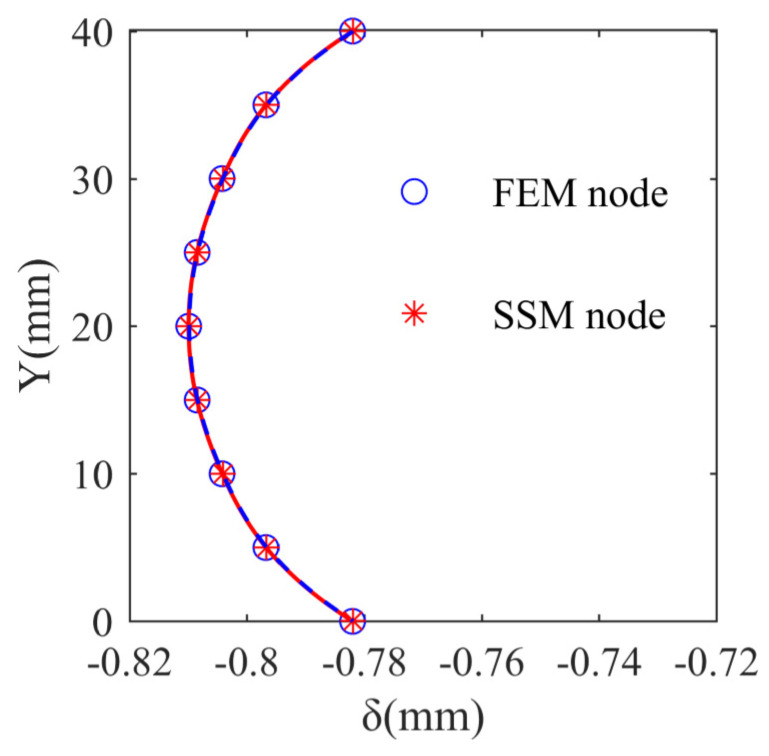
Comparison of SSM and traditional FEM results.

Figure 14 shows a comparison of the prediction results of the two methods. The prediction results of the two methods were consistent, which indicates that the prediction accuracy of the SSM is not affected and that it is capable of achieving the same accuracy as the prediction model based on traditional FEM.

**Figure 15:**
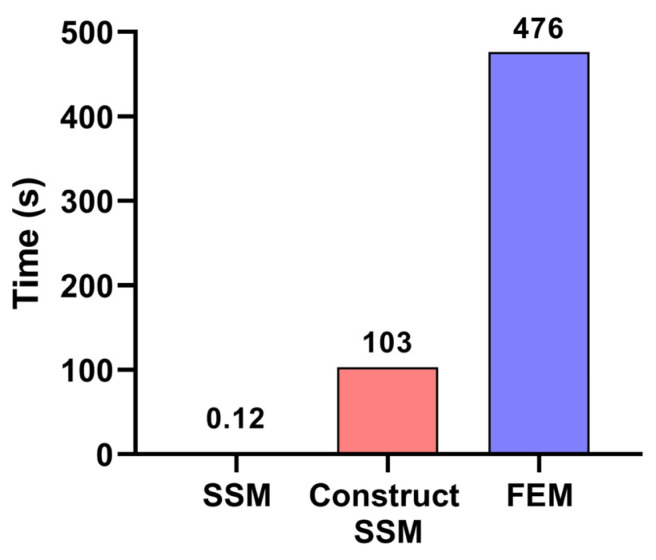
Comparison of computational efficiency of SSM and FEM.

To compare the computational efficiency of the two methods, a comparison graph of the time taken by the two methods for deformation prediction was plotted as shown in Figure 15. The time spent on the SSM model construction is also shown, and it is different from the calculation process of deformation prediction because it does not require the iterative calculation of the coupling between force and deformation.

As shown in Figure 15, the deformation prediction process based on SSM only required 0.12 s, despite the 103 s that were required for its establishment. The deformation prediction process based on traditional FEM took approximately 476 s, indicating that the computational efficiency of the traditional FEM is significantly less than that of the proposed SSM-based prediction method. Even if the establishment of SSM is included as part of the SSM-based deformation prediction process, the time consumed by FEM-based deformation prediction is still approximately 4.6 times that of the SSM-based method. This result shows that the proposed SSM method is significantly better than the FEM-based method in terms of computational efficiency.

Moreover, when the machining parameters must be changed, the traditional FEM prediction model must be re-simulated, which is a time-consuming task. However, for the SSM-based method, changing the machining parameters would not affect the prediction process of the established SSM model. The SSM can continue to be used and need not be re-established. Thus, compared to FEM-based methods, the SSM-based method is more flexible and efficient.

### 5.3. Verification of SSM Deformation Prediction Effect

**Figure 16:**
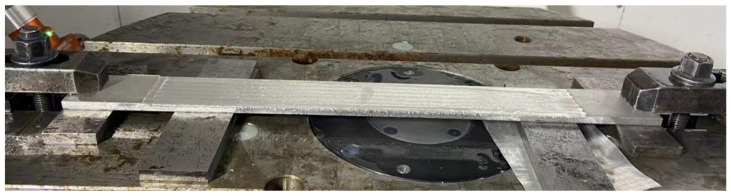
Clamping state during actual machining of thin plate.

To analyze the impact of the prediction model, uncompensated machining tests were conducted, and the predicted deformation results were compared with those of the actual machining effect. However, this part produced severe chatter during actual machining, resulting in the failure of normal machining. This result may be attributed to the fact that the thin plate part was too long, and the local stiffness in the middle of the part was too low. This effect may be also caused by the resonance due to the proximity of the cutting vibration frequency to the natural frequency of the part. As shown in Figure 16, to solve this problem, we added two pads on both sides of the thin plate part to improve the local stiffness of the part and suppress the impact of the flutter.

The addition of pads may decrease the computational accuracy of the prediction model. However, the models for complex parts are often simplified in actual simulation modeling, and thus, local simulation prediction deviations are unavoidable. Moreover, the adaptive correction of the local deviation of the simulation prediction model is also an important issue that should be considered in the iterative optimization compensation method proposed herein. In this experiment, the pads were introduced to verify the application effect of the proposed method. Therefore, the SSM is not rebuilt according to the parameter information of the pads. In the subsequent compensation machining process, the SSM without consideration of the pads was followed.

**Figure 17:**
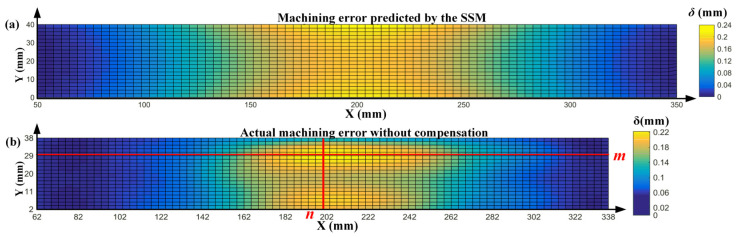
Machining result of the first layer of the part: (**a**) prediction results based on simulation, and (**b**) actual uncompensated machining results.

Figure 17a shows the deformation prediction result of the first layer of the part based on SSM, and Figure 17b shows the measured machining error distribution of the first layer after uncompensated machining. From Figure 17a, it can be observed that the predicted part machining deformation errors are symmetrically distributed along the center line, the maximum error occurs at the upper and lower ends of the vertical centerline, and the maximum error is 0.24 mm. Comparing Figure 17b with Figure 17a shows that the overall deformation trend along the length of the part is consistent between the actual measurement and predicted results. They all have small machining errors near the clamping positions on both sides, with the largest machining errors near the middle position. However, comparing specific local details shows that there are several differences between the actual machining and the predicted results. First, the actual machining errors shown in Figure 17b are not symmetrically distributed along the center line. Moreover, on the upper edge of the part, the area where the tool gradually moves away from the workpiece, the actual machining error is significantly smaller, and this area possesses the smallest machining error along the width direction. The maximum machining error along the width direction does not appear at the upper or lower edges of the part but at two positions that are located 10 mm from the edges on both sides.

The comparison results shown in Figure 17 reveal that despite the impact of the pads, the prediction results solely based on SSM are consistent with the actual machining results in the overall trend. However, several differences exist between the two results in terms of specific error distribution. The distribution of the errors in actual machining is more complicated, and the errors increase further with the machining depth. These deviations cannot be ignored because compensation machining directly based on prediction results may lead to excessive error.

### 5.4. Validation of the Proposed Iterative Optimization Compensation Strategy

To verify the effectiveness of our proposed iterative optimization compensation method combining OMM and SSM, we designed three sets of experiments. Among them, group 1 was the uncompensated machining experiment mentioned in Section 5.3 as the control group. The other two groups were the experimental groups applying our proposed method and were slightly different. In group 2, the iterative optimization compensation machining based on the SSM was used for the first time; in group 3, the iterative optimization compensation machining based on the optimized SSM of group 2 was used.

For a detailed comparison of the iterative optimization and compensation effects of each cutting layer in the machining process, cross-sectional curves were adopted to show the error distribution of the prediction model. As shown in Figure 17b, the two sections with the largest actual machining errors along the length and width directions, the m-section line, 10 mm from the upper edge, and the n-section line, where the vertical centerline is located, were chosen as the comparison sections for the machining results.

**Figure 18:**
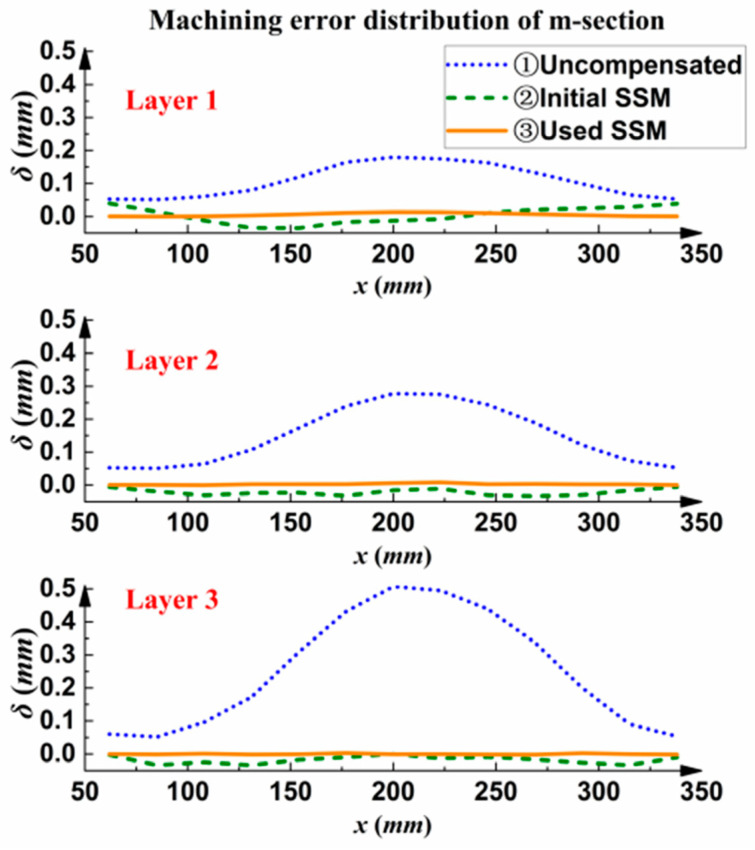
Measured machining error distribution of experimental parts in the m section.

After machining, the actual measurement data of the m section were used to establish the measured machining error distribution diagram shown in Figure 18.

As shown in Figure 18, with the uncompensated machining method, the machining error of the part increased gradually as the cutting layer deepened, and the machining error of the third layer reached close to 0.5 mm. This value is outside the tolerance of the part. The iterative optimization compensation machining method based on the initial SSM had a large error in the machining of the first layer; the maximum error was 0.052 mm. With increasing cutting layer depth, the machining error decreased gradually, and the final error was less than 0.05 mm, which meets the machining accuracy requirements of parts. By using the SSM iterative optimization compensation machining method, the machining error from the first layer to the third layer was maintained within 0.02 mm.

**Figure 19:**
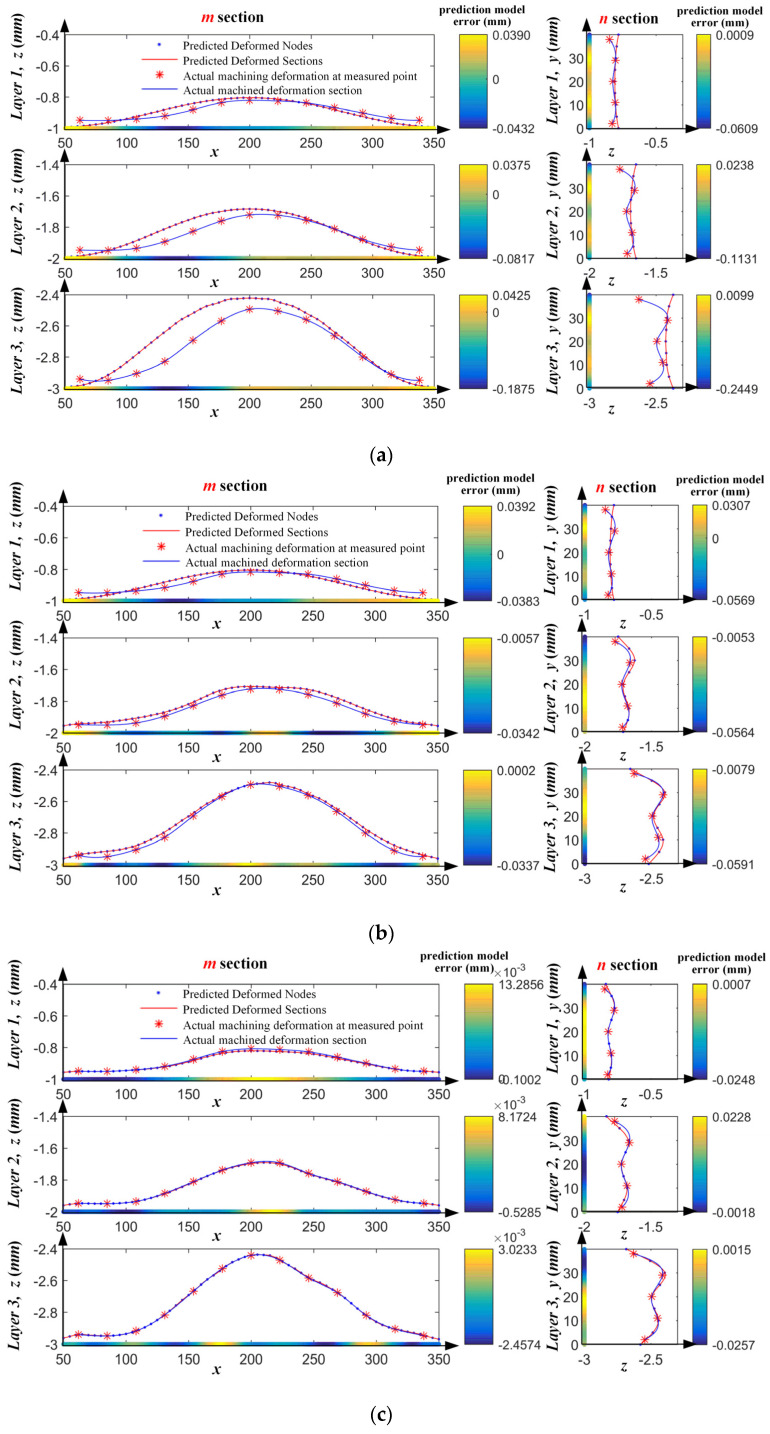
Analysis of prediction results based on SSM. (**a**) Machining without compensation; (**b**) iterative optimal compensation machining based on the initial SSM; (**c**) iterative optimal compensation machining based on the used SSM.

To analyze the iterative optimization process of the SSM model based on OMM results, the deviation analysis diagram of the prediction model as shown in Figure 19 was established. In the figure, the left area is the section parameter curve of the m section, and the right area is the section parameter curve of the n section. The red asterisk points on the blue curve indicate the actual deformation amount of the part at the measurement points, obtained by adding the compensation amount of the measurement points’ position to the errors measured after machining. The black dots on the red curve represent deformed nodes predicted by SSM. The colored straight line is the distribution diagram of the deviations of the above two curves, i.e., the deviation distribution diagram of the prediction model.

Figure 19a shows an analysis diagram of the uncompensated machining process. The compensation amount during machining was zero, and the actual measurement result was the deformation amount during the machining of the part. It is evident from the figure that with increasing cutting layer depth, the machining error increased gradually, and the maximum error in the last layer was approximately 0.5 mm. The uncompensated machining can no longer meet the requirements of machining accuracy. The colored line plot in the figure represents the deviation of the predicted deformation from the actual machining deformation of the part. Similarly, with increasing cutting layer depth, the deviation of the prediction model gradually increased, reaching 0.2449 mm in the last layer. This result indicates that simply performing compensation machining according to the simulation prediction results still cannot meet the machining accuracy requirements.

Figure 19b shows the results of the iterative optimization compensation machining experiment based on the initially used SSM. Because the SSM was applied for the first time, the initial values of the correction coefficients *α*_*j*,*i*_ and *β*_*j*,*i*_ were both 1. Moreover, because of the existence of the pads, compensation machining according to the prediction model resulted in local overcut in the areas of the pads, resulting in poor compensation for the first layer, as shown in Figure 19b. However, through iterative correction of the prediction model along the depth direction based on *β*_*j*,*i*_, the errors of the second and third layers of the part were significantly reduced. After iterative correction, the maximum error of the prediction model after the compensation machining of the third layer was reduced to −0.0591 mm, which sufficiently met the accuracy requirements of the part. Compared with the prediction results based solely on SSM, the error of the prediction model was reduced by 75.9%, which shows that the proposed method can be potentially used in the compensation of machining of single parts.

Figure 19c also shows the result of compensation machining based on the proposed method. The main difference is that the machining was based on the optimized SSM of the experimental results of group 2. Compared with the results of compensation machining based on the initially used SSM in the first layer as shown in Figure 19b, the error of the prediction model was significantly reduced, exhibiting a drop of 56.4%, with the maximum error reduced from −0.0569 mm to −0.0248 mm. Compared with the results shown in Figure 19b, the prediction model errors in the second and third layers were lower and less fluctuating. which indicates that the prediction accuracy of SSM can be significantly improved by iterative correction based on the correction coefficient between parts *α*_*j*,*i*_.

## 6. Conclusions

In this study, an SSM containing discrete node stiffness information, which can be used for fast machining deformation prediction, was established. The model exhibited higher flexibility and faster iterative calculation speed. An iterative optimization and compensation method for machining deformation of thin-walled parts based on OMM and SSM was proposed and was validated through end milling experiments of thin-walled plates with pads added. From the experimental results, the following conclusions can be drawn:

(1) Compared to the pure FEM-based machining deformation prediction model, the proposed SSM model exhibits higher computational efficiency and flexibility while ensuring essentially the same prediction accuracy.
(2) For the initially used SSM, iterative correction based on the interlayer correction coefficient *β*_*j*,*i*_ can gradually improve the accuracy of compensation machining, The deviation of the prediction model that was iteratively optimized by OMM was reduced by 75.9%. Thus, the workpieces can finally meet the accuracy requirements, indicating that the proposed method can be used for the compensation of single parts.
(3) For parts that are machined in batches, the iterative correction based on the inter-part correction coefficient *α*_*j*,*i*_ can further improve the accuracy of compensation machining, which is 56.4% higher than that of SSM used for the first time. The utilization rate of machining error information is improved through the use of *α*_*j*,*i*_ and *β*_*j*,*i*_.

## Figures and Tables

**Table 1:** Material parameters of thin plate parts.

Material	Young’s Modulus (GPa)	Poisson’s Ratio	Density (kg/m^3^)
AL6061	70	0.33	2.75 × 10^3^

## Data Availability

Data are contained within the article.
